# Combined metabolome and transcriptome analysis provides molecular insights into reproductive process in Chuanxiang Black and Landrace pigs

**DOI:** 10.3389/fgene.2025.1501876

**Published:** 2025-02-28

**Authors:** Jiangling Li, Jinling Zhang, Sujun Zhao, Qiushi Wang, Rui Liu, Xiaohui Chen, Zhiping He

**Affiliations:** Animal Breeding and Genetics Key Laboratory of Sichuan Province, Sichuan Animal Science Academy, Chengdu, Sichuan, China

**Keywords:** transcriptome, metabolome, testis, DEGs, landrace pigs, Chuanxiang Black pigs

## Abstract

Testes are crucial for male reproduction, and transcriptomic and metabolomic analyses can help identify genes and pathways linked to reproductive performance differences in pig breeds. The present study was conducted to identify the differentially expressed genes and differentially accumulated metabolites (DAMs) through transcriptomic and metabolomic analyses of testicular tissues in Chuanxiang Black and Landrace pigs. Six testis tissue samples from each pig breed were used for transcriptomic analysis. Further liquid chromatography-mass spectrometry analysis was performed for targeted metabolomic analysis to identify differential metabolites in both breeds. RNA-sequencing data identified a total of 6,233 DEGs, including 3,417 upregulated and 2,816 downregulated genes in Chuanxiang Black compared to Landrace pigs. Comparative pathway enrichment analyses revealed that many DEGs and DAMs were associated with critical reproductive pathways, especially those related to male gametogenesis, spermatogenesis, sexual reproduction, development, and reproductive processes. Three major pathways related to signal transduction (PI3K-Akt, Rap1, and MAPK signaling pathways), lipid metabolism (linoleic acid and arachidonic acid metabolism), and cytokine-cytokine receptor interaction were identified as differentially enriched pathways in Chuanxiang Black pigs. Differential circRNA target gene enrichment analysis revealed 4,179 DEGs, including 3,022 genes involved in biological processes, 477 in cellular components, and 680 in molecular functions. Differential analysis of miRNA between the two groups revealed 2,512 DEGs, including 1,628 upregulated and 884 downregulated genes. Both miRNA and circRNA were involved in enriched KEGG pathways mainly including signaling pathways (cAMP signaling pathways, calcium signaling pathways), endocrine secretion (aldosterone synthesis and secretion and GnRH secretion), and signaling molecules and interaction (ECM-receptor interaction). These findings revealed that both circRNA and miRNA play a crucial role in regulating the differential gene expression related to reproductive processes in Chuanxiang Black compared to Landrace pigs.

## 1 Introduction

Landrace (L) is one of the most widely used commercial pig breeds, which is known for its large size and high meat yield, with significant contribution to the pork industry (Guan et al., 2023). They are famous for their rapid growth, lean meat, and good maternal qualities, which make them a preferred choice in crossbreeding programs for commercial pork production (Knap, 2022). In contrast, Chuanxiang black (S) pig is a new breed in China that has been developed by crossing Tibetan and Duroc pig breeds. The Chuanxiang black pig breed has completely black fur, high growth efficiency, and excellent meat quality (Liu et al., 2023). The basic objective of the development of this breed was to combine the growth potential of commercial exotic pigs with the adaptability characteristics of indigenous breeds to ensure sustainable global food security, genetic diversity, and to address diverse consumer preferences in the pork industry (Towers, 2016; Liu C. et al., 2023; Liu H. et al., 2023).

The testes are major organs of the male reproductive system, primarily responsible for androgen production, spermatogenesis, and sperm maturation (Gurung et al., 2022). In different pig breeds, the efficiency of these processes varies significantly, influencing overall reproductive performance (Towers, 2016). To understand variability in reproductive efficiency and its underlying mechanism, it is imperative to study transcriptomic and metabolomic profiles of testicular tissue of diverse pig breeds varying in reproductive efficiency (Wagner et al., 2023). Such analyses can unravel the complex interplay of major genes and pathways that differentiate reproductive capabilities among pig breeds (Feng et al., 2023).

It is well established that male fertility depends on the efficacy of spermatogenesis, which is a complex process precisely controlled by fine regulation during which spermatogonium finally develops into mature sperm through continuous mitosis, meiosis, and cell differentiation (Cai et al., 2019; Cannarella et al., 2020). It is well-documented that different genes regulate the process of spermatogenesis in the developing testis. About 1,652 genes associated with spermatogenesis have been identified, out of 351 were expressed only in the male germ cells, with germ cell-specific transcripts being much less common earlier in testicular development (Schultz et al., 2003). Expression of some of these genes is inevitable for normal sperm formation like *ADAM2* whose absence can result in abnormal sperm functions (Cho et al., 1998). Similarly, normal functioning of *Tnp2* gene is required for sperm maturation and fertility (Tseden et al., 2007). Moreover, small RNAs like miRNA and circRNA can regulate the targeted genes that are involved in mammalian testicular development and spermatogenesis (Li et al., 2016). Therefore, it is imperative to investigate the differential genes, differentially accumulated metabolites, and associated non-coding RNAs to reveal differences in the reproductive capacity of different pig breeds. The present study aimed to investigate the intricate biological landscape by comparing DEGs and DAM of testicular tissue from two distinct pig breeds (Chuanxiang Black and Landrace).

## 2 Materials and methods

Male pigs of Chuanxiang Black (S) and Landrace breeds (L) raised under the same environment and management conditions were used for this study. L reaches sexual maturity at 6 months of age and can be mated at 8 months, while S reaches sexual maturity at 5.5 months of age and can be mated at 7 months. Six tissue samples of testes from Landrace (L01, L02, L03, L04, L05, and L06) and Chuanxiang Black (S01, S02, S03, S04, S05, and S06) pigs were collected.

### 2.1 Tissue sampling, and metabolite extraction

A total of 12 testicular tissue samples were collected from each breed at 14 days of age following castration and were immediately placed in liquid nitrogen for cryopreservation. Briefly, about 10g testicular tissue sample was ground with 100 mL of liquid nitrogen and transferred to an Eppendorf tube, and added 500 μL of 80% methanol. Then mixture was vortexed and put in the ice box for 5 min. After that, the homogenate was centrifuged at 15,000 *g* (4°C) for 20 min. After centrifugation, supernatant plus mass spectrometry grade water was added to dilute it to a methanol content of 53% and stored at −20°C overnight. The tissue extracts were then centrifuged at 15,000 *g* at 4°C for 20 min and 400uL supernatant was injected for Liquid chromatography-mass spectrometry analysis to conduct targeted metabolomic analysis on the highly sensitive SCIEX QTRAP^®^ 6500+ mass spectrometry platform (SCIEX, Framingham, United States).

### 2.2 OPLS-DA analysis for screening of differential metabolites

Raw MS spectra were processed using XCMS package, which included steps for peak detection, alignment, and retention time correction. Peaks were identified based on m/z ratios and retention times, following established protocols (Smith et al., 2006). The metabolome data were processed and analyzed using MetaboAnalyst 4.0 (Chong et al., 2018), which supports detailed statistical and pathway enrichment analyses. It was used to perform LOG transformation and Par-scaling formatting processing on the metabolome data, and the first principal component was modeled and analyzed by orthogonal partial least squares discriminant analysis (OPLS-DA) as described previously (Chong et al., 2018). The input data for MetaboAnalyst consisted of a feature matrix derived from processed MS spectra, including m/z ratios, retention times, and peak intensities. Data preprocessing included log transformation and Pareto scaling to minimize the impact of large variations. The Variable Importance in the Projection (VIP) value of the PLS-DA model and the p-value of the independent sample T-test were used to identify differentially accumulated metabolites (DAMs) using differential metabolite screening threshold: VIP≥1 > P-value≤0.05 > FC ≥ 2. The KEGG and PubChem databases were used to perform pathway enrichment analysis of differential metabolites between both pig breeds.

### 2.3 Total RNA extraction and sequencing

Total RNA extraction from testis tissue samples was performed using the RNeasy Midi Kit (Qiagen, Germany). The quantity of RNA was measured by a NanoDrop ND-1000 Spectrophotometer (NanoDrop Technologies, DE, United States), and the quality was assessed through the RNA 6000 Nano LabChip^®^ Kit on 2100 Bioanalyzer (Agilent Technologies, CA, United States). About 1 μg of RNA was used to construct the sequencing library by using the Illumina TruSeq RNA sample preparation kit (Illumina, San Diego, CA, United States) as described previously (Srikanth et al., 2017). The sequencing was performed on the Illumina Novaseq sequencing platform using pair-end sequencing (150 bp).

### 2.4 Analysis of RNA-Seq data

The RNA-seq reads were aligned to the *Sus scrofa* 11.1 reference genome using HISAT2 (Srikanth et al., 2019). Genome annotations were obtained from Ensembl to ensure accurate mapping and quantification of transcripts. Briefly, raw reads were processed using FastQC (version 0.11.5) for quality control (Andrews, 2017) followed by the use of TRIMMOMATIC (version 0.36) for trimming of adaptors and low-quality bases (Bolger et al., 2014), and alignment with pig reference genome (*S. scrofa* 11.1) using HiSAT2 (ver. 2.05) software (Kim et al., 2019). The aligned reads were enumerated through Feature Counts (version 1.5.0) as described previously (Liao et al., 2014). After eliminating batch effects and noise using Svaseq (Leek, 2014), the analysis of differentially expressed genes (DEGs) was conducted through DESeq2 (Love et al., 2014). After the determination of significant DEGs (FDR < 0.1), functional enrichment analysis was performed through Gene Ontology using DAVID (Huang et al., 2009). Further, pathway enrichment analysis using Kyoto Encyclopedia of Genes and Genomes was conducted through KOBAS (http://kobas.cbi.pku.edu.cn/kobas3/). Identification of circRNA was conducted using CIRI2 (Gao et al., 2015), which detects back-splice junctions from RNA-seq data. miRNA prediction and quantification were performed using miRDeep2 (Friedländer et al., 2012), leveraging the pig miRNA database for accurate annotation.

## 3 Results

### 3.1 Identification of differentially expressed genes

Twelve cDNA libraries were constructed and sequenced for the transcriptome analysis of Chuanxiang Black and Landrace pigs. Supplementary Table S1 provides sequencing statistics, and Supplementary Figure S1 shows the overall distribution of FPKM values, indicating consistent sequencing quality across Chuanxiang Black and Landrace pig samples. However, these metrics alone do not establish similarities or differences in gene expression patterns between the breeds.

**FIGURE 1:**
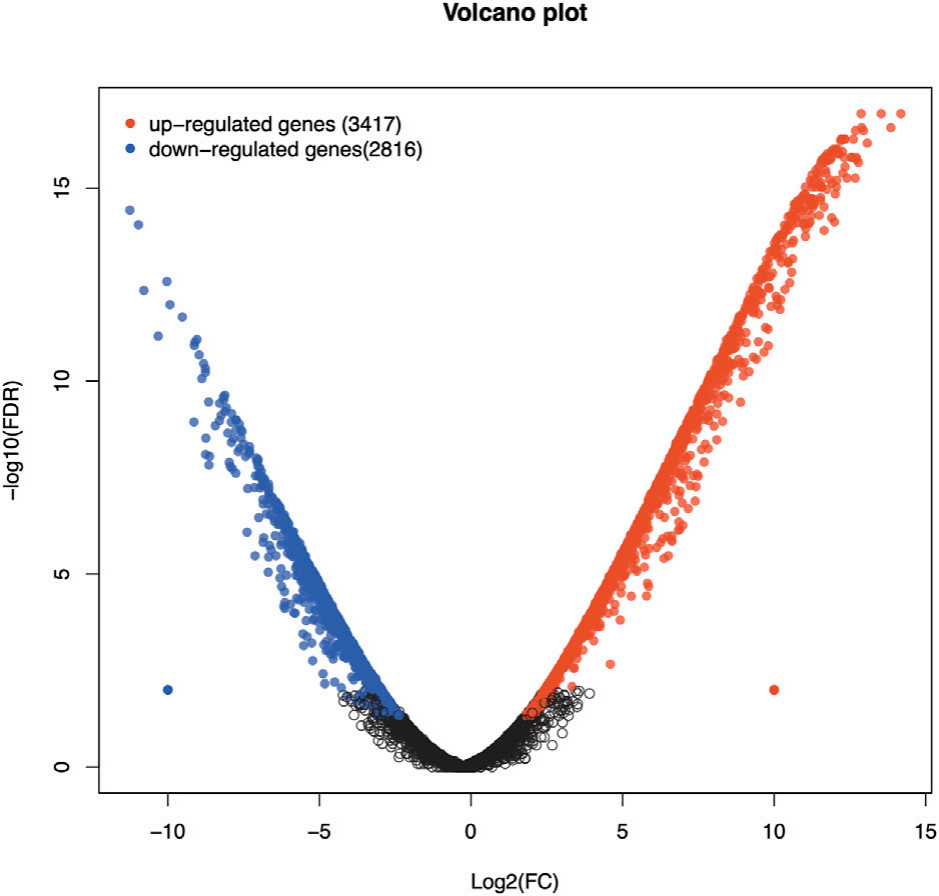
Volcano plots of Volcano plot of differentially expressed genes in Chuanxiang Black compared to Landrace pigs. Red, black, and blue represent upregulated, not differentially expressed, and downregulated genes. The x-axis represents the fold change in gene expression, while the y-axis represents the statistical significance of the discrepancies.

Analysis of DEGs revealed a total of 6,233 genes including 3,417 upregulated and 2,816 downregulated genes in Chuanxiang Black pigs compared to Landrace (Figure 1). The top 10 DEGs are presented in Table 1.

**TABLE 1:** List of top 10 differential genes description.

Gene ID	Gene name	Type	P Value
TBP	TATA-box-binding protein	up	2.57E-05
DLL1	delta-like protein 1 isoform X1	down	3.36E-05
TCTE3	tctex1 domain-containing protein 3	up	7.09E-11
THBS2	thrombospondin-2 isoform X1	down	0.00225
SMOC2	SPARC-related modular calcium-binding protein 2 isoform X1	down	0.0149
DACT2	dapper homolog 2	down	0.00126
KIF25	kinesin-like protein KIF25 isoform X1	up	0.00304
LOC106506286	uncharacterized protein LOC106506286 isoform X1	down	0.00010
UNC93A	protein unc-93 homolog A isoform X1	up	0.00186
TTLL2	tubulin polyglutamylase TTLL2	up	0.00034

### 3.2 GO term and KEGG pathway analyses for DEGs

**FIGURE 2:**
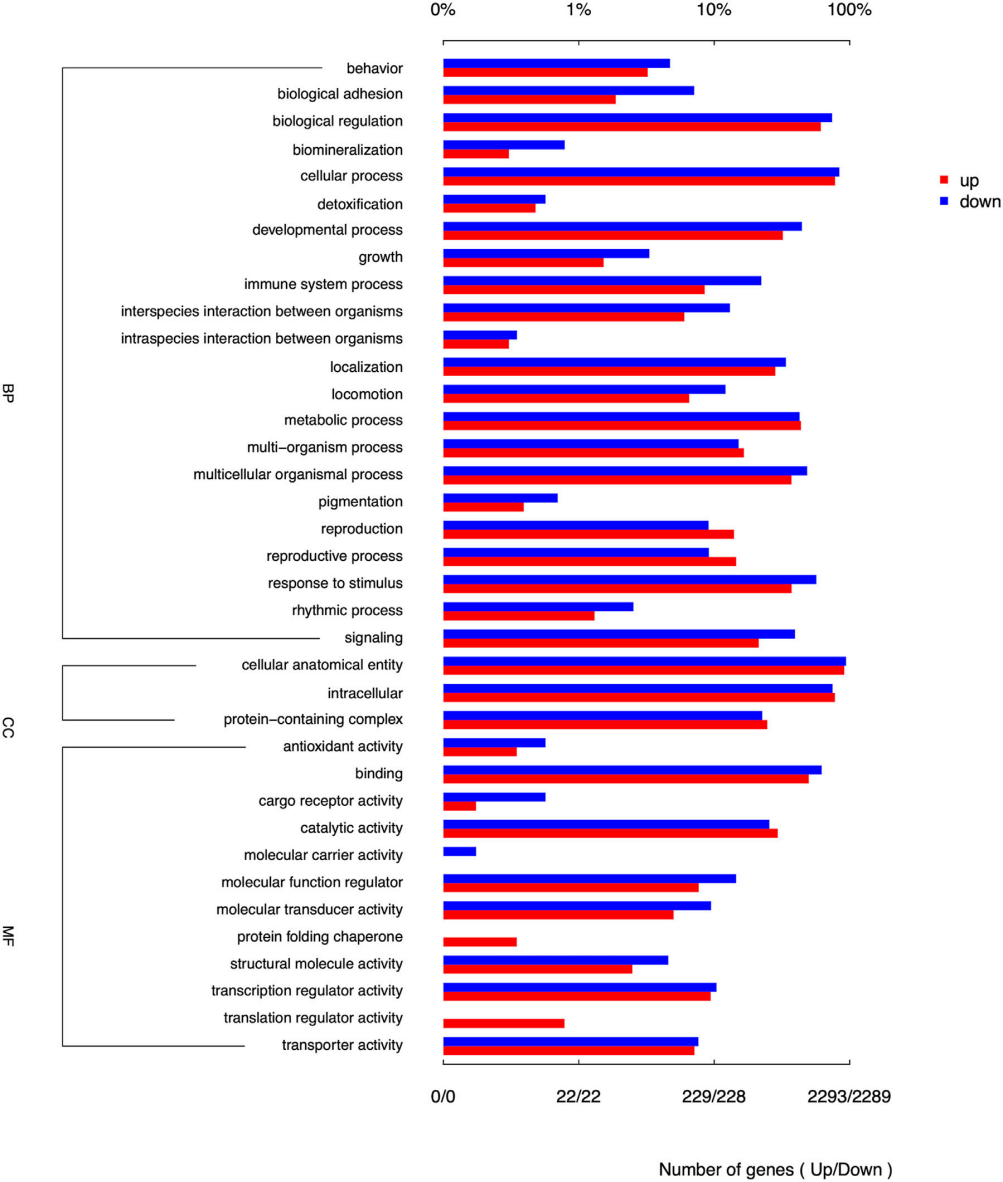
Enrichment of GO terms of DEGs for biological process (BP), cellular components (CC) and molecular functions (MF) in Chuanxiang Black compared to landrace pigs.

Among the top 10 DEGs analyzed, *TBP, TCTE3, KIF25, UNC93A*, and *TTLL2* genes showed upregulation, indicating increased expression levels. Conversely, *DLL1, THBS2, SMOC2, DACT2,* and *LOC106506286* genes exhibited downregulation. The pathway enrichment of DEGs in Chuanxiang Black was compared to Landrace pigs using gene ontology (GO) and KEGG pathway analyses (Figure 2). The differential genes exhibited notable enrichment in biological process, behavior, biological adhesion, biological regulation, biomineralization, cellular process, detoxication, growth, immune system process, localization, metabolic process, and multi-organism process. The DEGs in the cellular component category were involved in cellular anatomical entity, intracellular process, and protein-containing complex. The predominant enrichment keywords in molecular function were primarily related to the protein folding chaperone, translation regulator activity and the activity of structural molecules.

**FIGURE 3:**
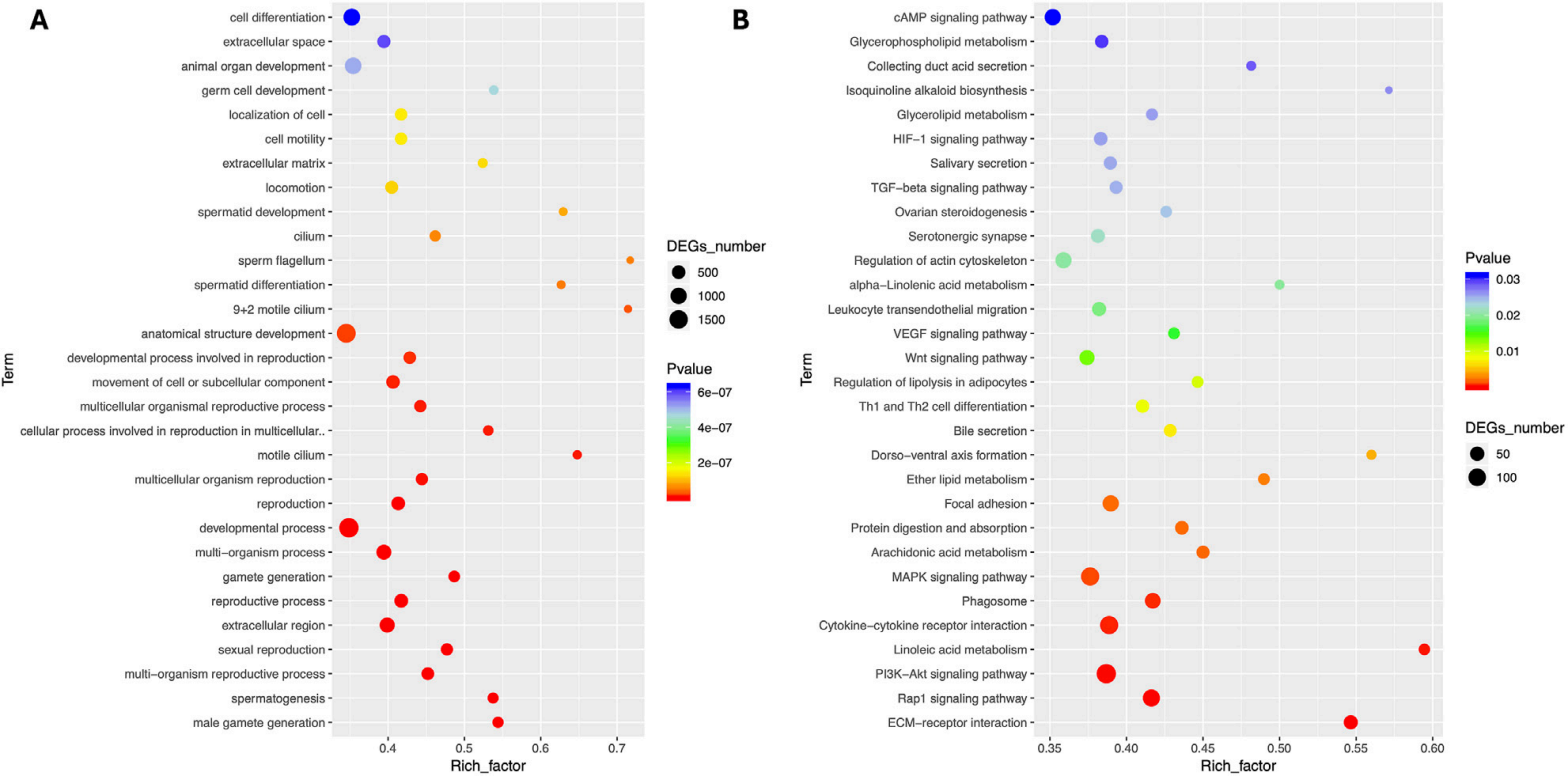
Comparative pathway enrichment analyses of Differentially expressed genes. **(A)** GO enrichment **(B)** KO enrichment between Chuanxiang Black compared to landrace pigs.

The comparative analysis of transcriptome results showed that a significant number of DEGs were associated with important reproductive pathways, particularly those involved in the formation of male gametes, spermatogenesis, sexual reproduction, development, and reproductive activities. GO enrichment analysis revealed major enriched pathways in Biological Process terms including cell differentiation, animal organ development, anatomical structure development, developmental process, gamete generation, gamete generation, reproductive process, sexual reproduction, multi-organism reproductive process, spermatogenesis and male gamete generation (Figure 3A). The KEGG enrichment analysis showed that major enriched terms including signal transduction (cAMP signaling, MAPK signaling, PI3K-Akt signaling and Rap1 signaling pathways), lipid metabolism (arachidonic acid metabolism), signaling molecules and interaction (cytokine-cytokine receptor interaction, and ECM-receptor interaction) and transport and catabolism (focal adhesion) as presented in Figure 3B.

**FIGURE 4:**
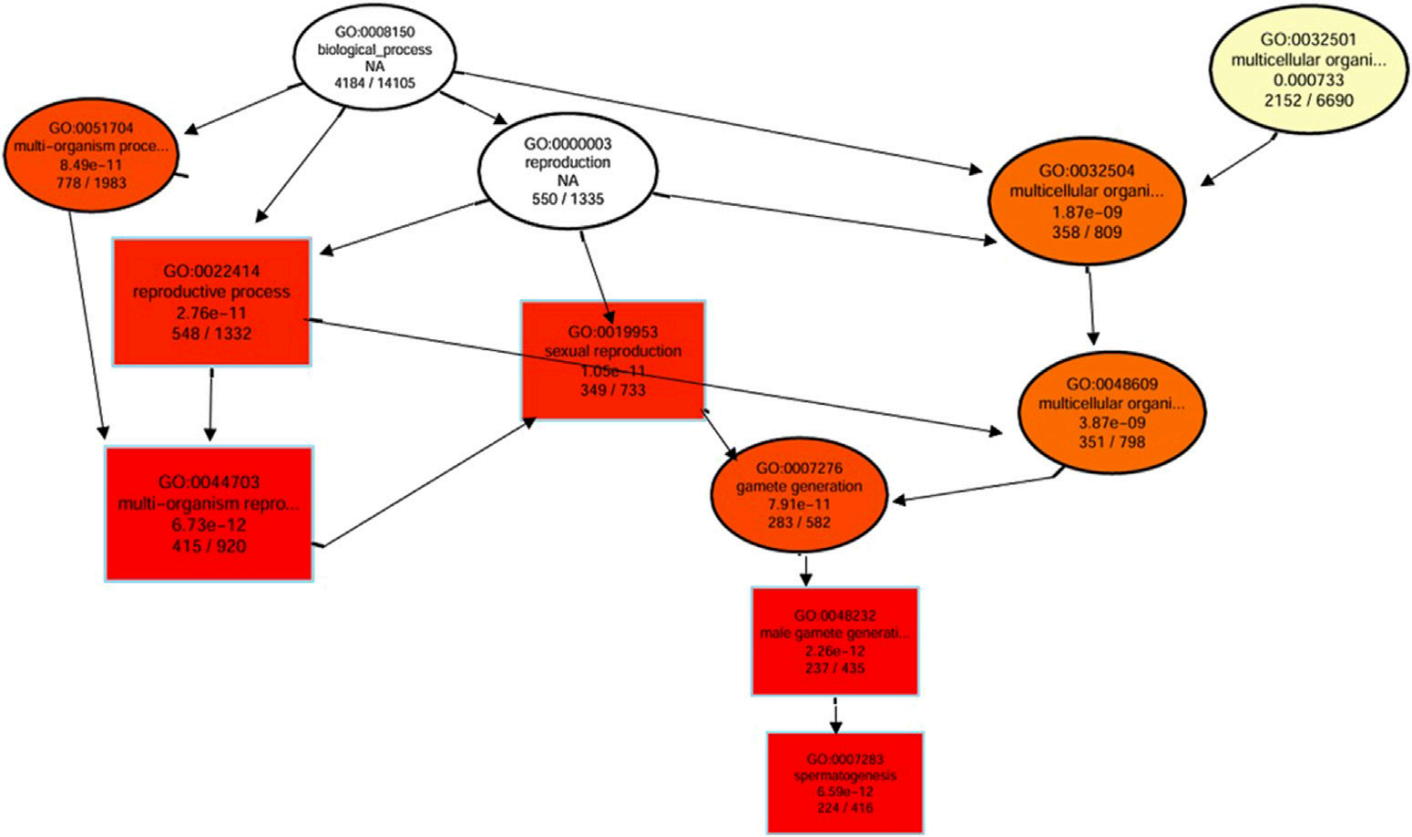
Directed acyclic graph (DAG) of the results of Gene Ontology (GO) enrichment of DEGs. The branches represent the containment relationships, and the range of functions decreases in size from top to bottom. Each node represents a GO term. The top 10 GO terms have been selected as the master nodes. A darker color indicates greater enrichment of the term. The name and p-value of each term are presented on the node.

A directed acyclic graph of the results of GO enrichment of DEGs was constructed to reveal the hierarchical relationship of GO terms in the biological process category (Figure 4). Results revealed that important biological processes interacted with each other to effectively regulate the reproductive process mainly affecting spermatogenesis. This indicated that DEGs in Chuanxiang Black pigs resulted in upregulation of the reproductive process especially spermatogenesis affecting fertility compared to Landrace pigs.

### 3.3 Metabolomic analysis

Differentially accumulated metabolites (DAMs) were identified in the testis tissues by comparing the metabolic profiles between Chuanxiang Black and Landrace pigs. A total of 755 metabolites were identified in the metabolomics analysis. Of these, 175 were identified as DAMs, including 93 upregulated and 82 downregulated metabolites in Chuanxiang Black compared to Landrace pigs, based on a Variable Importance in Projection (VIP) value ≥ 1, p-value ≤ 0.05, and fold change ≥ 2.

**FIGURE 5:**
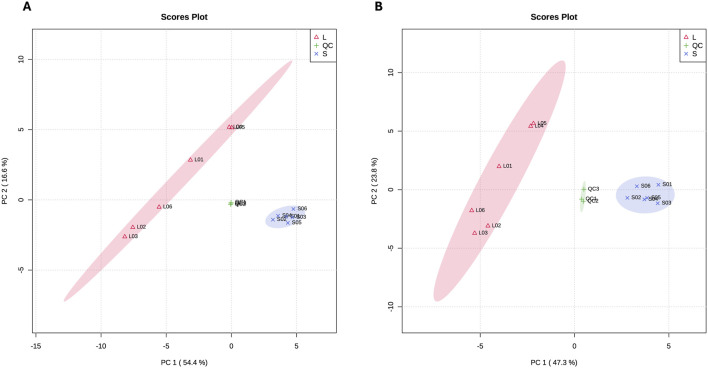
Principal Component Analysis (PCA) plots for metabolome data. **(A)** PCA conducted on the negative ion mode, and **(B)** PCA conducted on the positive ion mode. ‘L' represents samples from Landrace pigs, “S” represents samples from Chuanxiang Black pigs, and “QC” represents quality control samples included to assess the reproducibility and stability of the mass spectrometry data. The clustering of QC samples demonstrates the reliability of the analysis, while the separation of L and S samples indicates distinct metabolic profiles between the two breeds.

**FIGURE 6:**
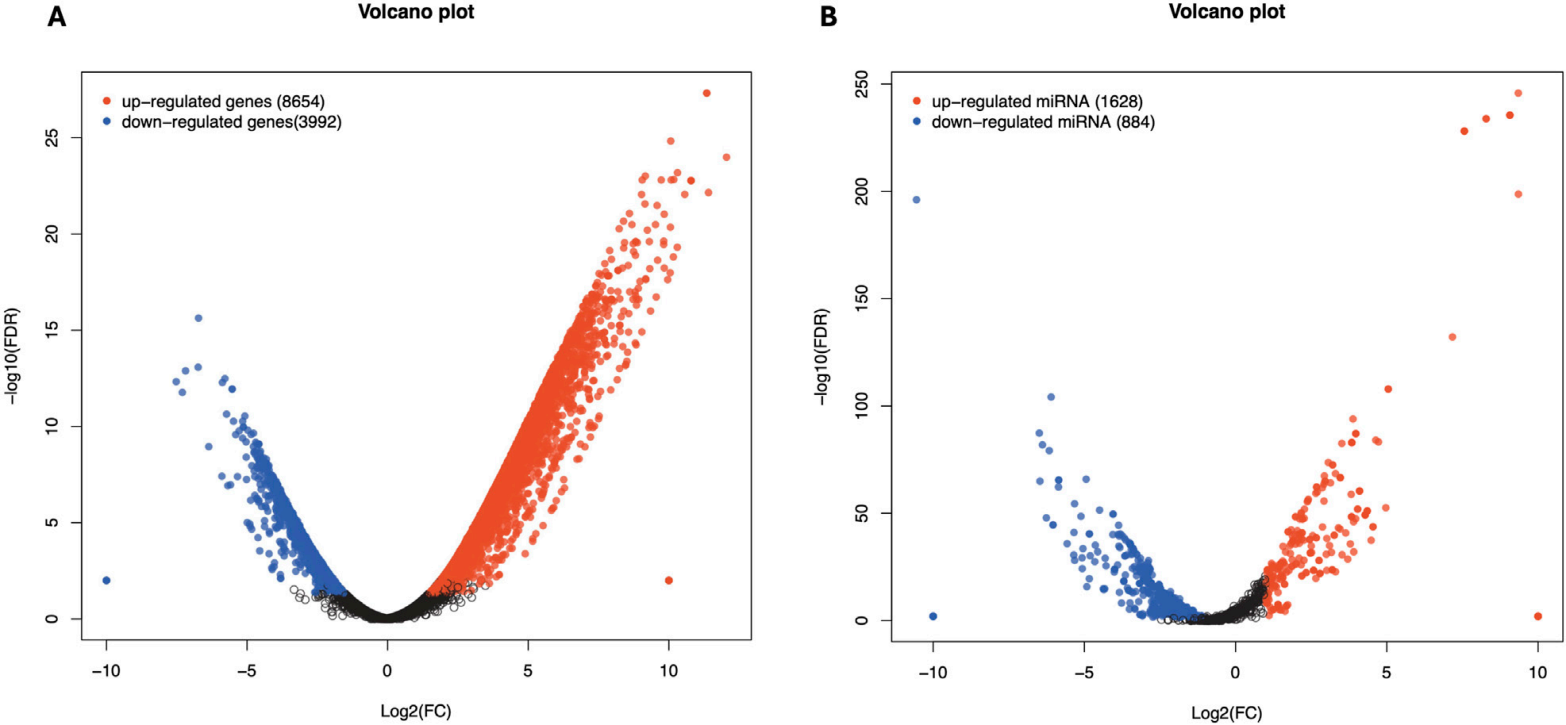
Differentially expressed genes in affected by cicrRNA **(A)** and miRNA **(B)** in Chuanxiang Black compared to Landrace pigs. Red, black, and blue represent upregulated, not differentially expressed, and downregulated genes. The x-axis represents the fold change in gene expression, while the y-axis represents the statistical significance of the discrepancies.

The top 10 DAMs in Chuanxiang Black compared to Landrace pigs are presented in Table 2. The two-dimensional principal component analysis score plots revealed distinct variations in the distribution of metabolites between Landrace and Chuanxiang Black groups in both the negative ion (Figure 5A) and positive ion models (Figure 5B). Additionally, the top 10 DAMs (6-Keto-prostaglandin F1alpha, Dihydroxyacetone phosphate, D-Glucose 6-phosphate, Methylmalonate, Tolfenamic acid, 5-Methoxytryptophol, 5′-Adenylic acid, 6-Sialyllactose, Succinic acid, and Riboflavin-5-phosphate, showed distinct variations between two pig breeds. Specifically, 6-Keto-prostaglandin f1alpha, Dihydroxyacetone phosphate, D-Glucose 6-phosphate, Methylmalonate, 5-Methoxytryptophol, and 6-Sialyllactose exhibited higher levels in Landrace pigs, whereas Tolfenamic acid, 5′-Adenylic acid, Succinic acid, and Riboflavin-5-phosphate showed higher levels in Chuanxiang Black breed. KEGG enrichment analysis of differential metabolites revealed that about five pathways were significantly enriched including signal transduction (cGMP-PKG signaling pathway and olfactory transduction), nucleotide metabolism (purine metabolism), endocrine system (thyroid hormone synthesis), and lipid metabolism (secondary bile acid synthesis) pathways (Figure 6).

**TABLE 2:** Top 10 differential Metabolites in Chuanxiang Black (S) compared to landrace (L) pigs.

Name	Formula	Landrace	Chuanxiang	log2(S/L)	P-value	VIP
6-Keto-prostaglandin f1alpha	C20H34O6	218,811.66	26,127.5	−3.06	0.011698	1.8601
Dihydroxyacetone phosphate	C3H7O6P	4,381,000	1,178,916.66	−1.89	2.41E-05	1.3492
D-Glucose 6-phosphate	C6H13O9P	77,215,000	19,345,000	−1.99	0.01123	1.1705
Methylmalonate	C4H6O4	523,400	2,446,300	2.22	0.003389	1.851
Tolfenamic acid	C14H12ClNO2	21,678.5	333,700	3.94	7.39E-07	2.9619
5-Methoxytryptophol	C11H13NO2	72,390	18,167	−1.99	0.0018895	1.5391
5′-Adenylic acid	C10H14N5O7P	99,405	275,366.66	1.46	0.023619	1.198
6-Sialyllactose	C23H39NO19	108,741.66	18,805	−2.53	5.63E-05	1.6817
Succinic acid	C4H6O4	939,200	4,430,333.33	2.23	0.0032409	1.8507
Riboflavin-5-phosphate	C17H21N4O9P	210,638.33	1,256,100	2.57	5.64E-05	2.025

### 3.4 Differential circRNA and miRNA target gene enrichment

**FIGURE 7:**
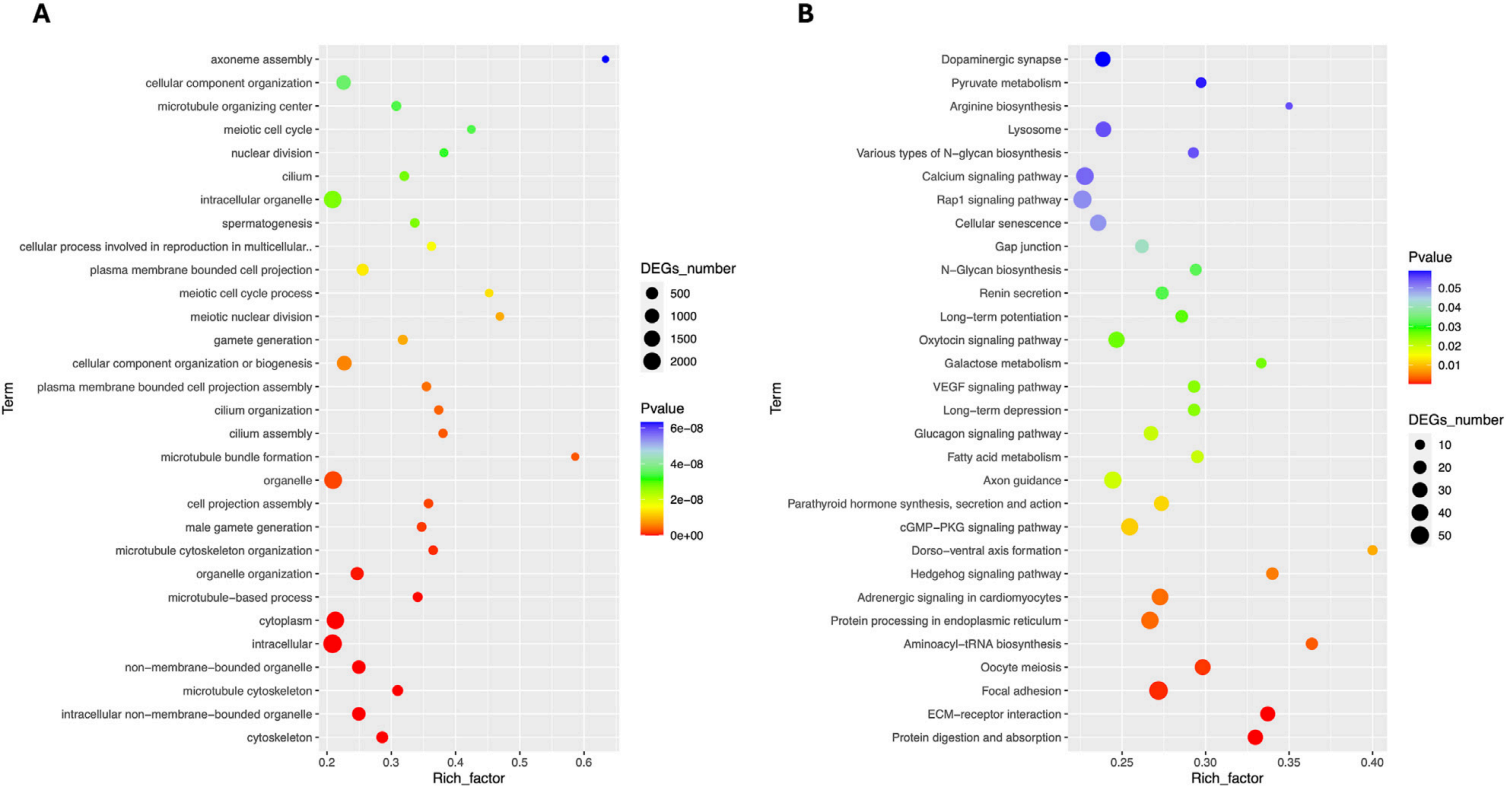
Gene ontology **(A)** and Kyoto Encyclopedia of Genes and Genomes **(B)** pathway analyses of differential circRNA in Chuanxiang Black compared to Landrace pigs.

**FIGURE 8:**
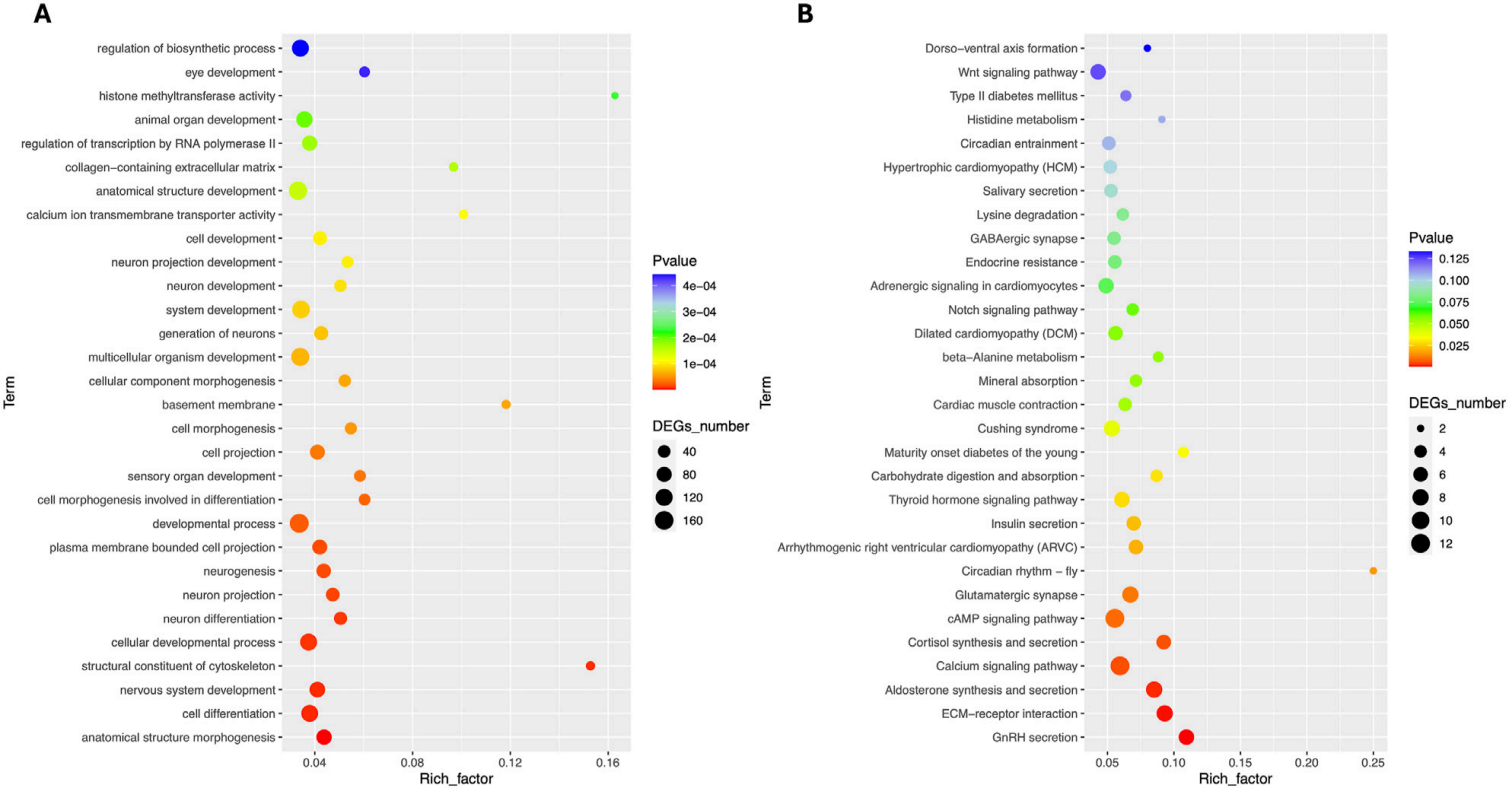
Gene ontology **(A)** and Kyoto Encyclopedia of Genes and Genomes **(B)** pathway analyses of differential miRNA in Chuanxiang Black compared to Landrace pigs.

Total RNA sequencing was performed, which included both coding and non-coding RNA. Small RNA analysis was conducted to identify circRNA and miRNA sequences from the total RNA-seq data, without requiring separate small RNA-seq libraries. Differential circRNA target GO enrichment analysis revealed 8,654 upregulated DEGs while 3,992 downregulated genes. The top 10 differential circRNA (showing highly significant differential expression in both breeds) along with their target genes are presented in Table 3. The distribution of differential circRNA in different samples of both pig breeds is presented in Supplementary Figure S2. The differential analysis of miRNA between two breeds revealed 2,512 DEGs including 1,628 upregulated and 884 downregulated genes. The top 10 miRNAs showing highly significant differential expression in both breeds and their target genes are presented in Table 4. These findings revealed that both circRNAs and miRNAs play a critical role in DEGs to regulate the reproductive process in Chuanxiang Black compared to Landrace pig breeds. GO enrichment analysis of differential circRNAs revealed several enriched GO terms in the Chuanxiang Black breed including cellular components, organelle, cytoplasm, intracellular organelle, cytoskeleton, gamete generation and spermatogenesis (Figure 7A). While KEGG pathways analysis revealed enriched pathways including signaling pathways (cGMP-PKG signaling, Rap1 signaling, oxytocin signaling and Glucagon signaling), parathyroid hormone synthesis, secretion and action, fatty acid metabolism, focal adhesion, and ECM-receptor interaction (Figure 7B). Similarly, pathway enrichment analysis of differential miRNAs revealed cellular developmental process, development process, nervous system development, cell differentiation and anatomical structure morphogenesis (Figure 8A). However, KEGG enrichment analysis revealed that differential miRNAs were involved in signaling pathways (cAMP signaling pathways, calcium signaling pathways), ECM-receptor interaction, Aldosterone synthesis and secretion, and GnRH secretion (Figure 8B).

**TABLE 3:** Top 10 differential circRNA target gene expression abundance and functional annotations in Chuanxiang Black compared to landrace pigs.

circRNA-ID	circRNA-region	Target-geneID	Effect	Gene name
circRNA55	NC_010443.5:857,884-870398	THBS2	up	Thrombospondin-2 isoform X1
circRNA63	NC_010443.5:1,530,120-1531521	KIF25	down	Kinesin-like protein KIF25 isoform X1
circRNA89	NC_010443.5:1,948,388-1952054	UNC93A	up	Protein unc-93 homolog A isoform X1
circRNA105	NC_010443.5:3238762-3324464	PDE10A	up	LOW QUALITY PROTEIN: cAMP and cAMP-inhibited cGMP 3′,5′-cyclic phosphodiesterase 10A
circRNA145	NC_010443.5:7130415-7166248	SLC22A3	up	Solute carrier family 22 member 3 isoform X1
circRNA146	NC_010443.5:7160497-7166248	SLC22A3	down	Solute carrier family 22 member 3 isoform X1
circRNA148	NC_010443.5:7375882-7384261	IGF2R	up	insulin-like growth factor II receptor
circRNA147	NC_010443.5:7375882-7378522	IGF2R	down	insulin-like growth factor II receptor
circRNA163	NC_010443.5:7593399-7598966	TCP1	up	T-complex protein 1 subunit alpha
circRNA164	NC_010443.5:7593510-7593793	TCP1	down	T-complex protein 1 subunit alpha

**TABLE 4:** Top 10 differential microRNA target gene expression abundance and functional annotations in Chuanxiang Black compared to Landrace pigs.

miRNA	Target gene	Gene Name/Function
miR-129	DBX1	homeobox protein DBX1
miR-148a-3p	NLRP12L	NACHT, LRR and PYD domains-containing protein 12 isoform X1
miR-199-5p	KPNA6	importin subunit alpha-7 isoform X2
miR-22c	EPB41L5	band 4.1-like protein 5 isoform X1
miR-27a	EHBP1	EH domain-binding protein 1 isoform X1
miR-27d-3p	EHBP1	EH domain-binding protein 1 isoform X1
miR-30b	TLR6	Toll-like receptor 6
miR-210	ANKRD50	Ankyrin repeat domain-containing protein 50
miR-33-5p	FSHR	Follicle-stimulating hormone receptor precursor
let-7c-1-3p	ARHGAP30	rho GTPase-activating protein 30 isoform X1

## 4 Discussion

The combined transcriptome and metabolome analysis of testes tissue of Chuanxiang Black and Landrace breeds identified several DEGs and metabolic pathways with crucial roles in reproductive processes. This study uniquely interprets genetic expression data along with metabolome profiles, offering a detailed view of the biological mechanisms underlying reproduction process in pigs. The findings are expected to enhance the understanding of breed-specific reproductive traits, potentially leading to improved breeding strategies and reproductive health in pigs.

In this study, we identified a total of 6,233 DEGs, including 3,417 upregulated and 2,816 downregulated genes in Chuanxiang Black compared to Landrace pigs. The pathway enrichment analyses of DEGs revealed a strong association of DEGs with critical reproductive pathways, particularly those involving male gamete synthesis, spermatogenesis, sexual reproduction, development, and reproductive processes. Additionally, KEGG analysis revealed that DEGs were associated with three major pathways including signal transduction (PI3K-Akt, Rap1, and MAPK signaling pathways), Lipid metabolism (Linoleic acid and Arachidonic acid metabolism) and Cytokine-cytokine receptor interaction pathways. The majority of DEGs were linked to the phosphoinositol-3-kinase (PI3K)/protein kinase B (AKT) signaling pathway. This pathway plays a crucial role in various aspects of male reproduction, such as controlling the hypothalamus-pituitary gonad axis during spermatogenesis, promoting the growth and specialization of spermatogonia, Sertoli and somatic cells, and regulating sperm autophagy and testicular endocrine function (Zhao and Yang, 2023). It is well established that endocrine hormones like follicle-stimulating hormone, estrogen, and thyroid hormone can regulate the PI3K/AKT signaling in Sertoli cells (Chen et al., 2022). In addition, PI3K/AKT signaling regulates proliferation and anti-apoptosis of immature Sertoli cells and spermatogonia. In addition, this signaling can collapse the structure of the blood-testis barrier through the synthesis of regulatory proteins and the cytoskeleton of mature Sertoli cells (Zhou et al., 2018). Taken together, all of the above effects mediated by PI3K/AKT signaling directly and indirectly promote and maintain spermatogenesis in the testis (Chen et al., 2022).

The second prominent pathway identified in the present study was the MAPK signaling pathway, which plays a crucial role in various male reproductive processes such as spermatogenesis, sperm maturation and activation, capacitation, and acrosome response (prior to egg fertilization). The MAPK signaling not only regulates the production of immature Sertoli cells but also determines their appropriate number in the testis at puberty, thereby sustaining fertility by ensuring the ability of males to produce sperms (Kumar et al., 2024). Moreover, MAPKs play a crucial role in maintaining cell-cell junctions in the testis, supporting germ cell proliferation and differentiation in Sertoli cells. They also regulate sperm fertility by meditating capacitation and the acrosome reaction in the female tract (Ni et al., 2019). Disruption of MAPK signaling can affect male fertility by impairing testicular homeostasis, sperm physiology, and sperm capacitation in the female tract during fertilization. The MAPK signaling pathway regulates the dynamics of tight junctions and adherens junctions, and the synthesis of lactate in Sertoli cells. Moreover, this pathway interacts with other pathways particularly TGF-β/Smad and PI3K/AKT/mTOR to regulate the dynamics of a complex regulatory network for spermatogenesis (Moreira et al., 2019; Ni et al., 2019). Similar findings were observed in the present study. In particular, mTOR within the PI3K/AKT/mTOR pathway is critical for the maintenance and differentiation of spermatogonial stem cells, as well as the regulation of redox balance and metabolic activity of Sertoli cells, which are essential for nutrient support during spermatogenesis (Chen et al., 2022; Deng et al., 2021).

Reproductive processes in the male pig are significantly influenced by the PI3K/AKT/mTOR signaling pathway. This signaling pathway plays a critical role in controlling numerous aspects of male reproduction, including the regulation of the hypothalamic-pituitary-gonadal axis during spermatogenesis and the growth and specialization of sperm progenitors and other supporting cells. Additionally, it contributes to the process of self-degradation of sperm cells and the hormonal activity of the testicles, particularly when exposed to environmental contaminants such as endocrine-disrupting chemicals (Jeng, 2014). Upregulation of the MAPK signaling pathway observed in Chuanxiang Black pigs compared to Landrace indicates superior fertility in boars of this breed. Such diversity in Landrace has been reported earlier as compared to the Duroc breed which is the parental breed of Chuanxiang Black pigs as Van Son et al., 2020 showed a significant variation in gene expression in testis tissue relating to sperm hyperactivity in Landrace boars. They identified 3,219 differentially expressed genes, the majority of which were upregulated in the context of spermatogenesis, as observed in the present study. They also identified overrepresented pathways among these DEGs by GO analysis, such as “extracellular exosome” and “cytoplasm”, which are essential for embryonic development (Van Son et al., 2020). This suggests a strong link between these pathways and reproductive efficacy.

The second most enriched term observed in pathway analysis was fatty acid metabolism. This differential expression suggests that linolenic and arachidonic acid metabolism pathways are altered between these phenotypic extremes. This indicates the importance of these pathways in the overall metabolic profile of pigs, which can indirectly influence reproductive health and function (Gol et al., 2019; Ramayo-Caldas et al., 2012). These findings highlight the complex interplay between genetic factors, lipid metabolism, and cytokine-cytokine receptor interactions in determining the reproductive capabilities and overall health of pigs. The role of these pathways in the reproductive processes underlines the intricate nature of the biological mechanisms governing reproduction in pigs.

The first top gene among the DEGs identified in this study was TATA box binding protein (TBP). This gene and its related family members play crucial roles in transcriptional regulation, which is essential for various biological processes, including reproduction. The TBP is involved in the assembly of transcription complexes at eukaryotic promoters and plays a key role in transcription initiation by forming part of several complexes involved in core promoter recognition and assembly of the preinitiation complex (Akhtar and Veenstra, 2011).

The second top differentially expressed gene identified in the present study was the T-complex-associated-testis-expressed 3 (*TCTE3*) gene which plays a significant role in reproductive processes, particularly related to sperm motility and morphology. Expression of *TCTE3* is significantly reduced in patients with asthenozoospermia and terato-asthenozoospermia, conditions characterized by reduced sperm motility and abnormal sperm morphology, respectively (Saberiyan et al., 2021). This suggests that *TCTE3* may have a direct influence on the flagella structure and function of sperm cells (Zhao et al., 2017). Moreover, a regulatory relationship between *TCTE3* and linc00574, a long non-coding RNA has been reported. The expression level of linc00574 was quite higher in patients with asthenozoospermia, and there was a positive correlation between *TCTE3* and linc00574 expression levels. These findings imply that *TCTE3* expression is modulated by linc00574 through a negative self-regulating mechanism (Saberiyan et al., 2021). This regulation is crucial for maintaining proper sperm motility and morphology, which are essential for male fertility. Therefore, the PI3K-Akt signaling pathway is likely the best pathway to consider when exploring the implications of the top 10 DEGS identified in testis tissues for fertility and reproductive efficiency in Chuanxiang Black compared to the Landrace breed.

Metabolomics enables the assessment of an organism’s physiological condition by tracking alterations in its internal metabolites. The present study aimed to identify and examine the overall biochemical variations in the reproductive processes in Chuanxiang Black and Landrace pigs. Metabolomic analysis identified the different enrichment of various metabolites observed in Chuanxiang Black compared to Landrace pigs in the present study. For example, the top metabolite identified was 6-Keto-prostaglandin F1 alpha (6-keto-PGF1 alpha), which is an important compound associated with reproductive processes in animals, particularly its role in ovulation and parturition is well established. It is reported that Prostagladins E2 (PGE_2_) in washed sperms of rams enhanced the level of sperm cAMP (Cosentino et al., 1982) but it is not clear whether it is a direct effect or receptor-mediated process. Prostaglandins can also regulate the Ca^2+^ uptake into the sperm cell by mediating intercellular cAMP levels (Hedqvist et al., 1980; Peterson et al., 1980). Moreover, it has been suggested that Prostaglandins are potentially involved in the transport of sperm in the distal epididymis and vas deferens (Cosentino et al., 1984). These studies suggest a potential role for Prostaglandins in spermatogenesis and male fertility. The KEGG enrichment analysis of differential metabolites revealed three major key pathways including signal transduction (cGMP-PKG signaling pathway and olfactory transduction), nucleotide metabolism (purine metabolism), endocrine system (thyroid hormone synthesis), and lipid metabolism (secondary bile acid synthesis) pathways. Major metabolites are related to signal transduction as diverse signaling pathway balances the anabolic and catabolic processes and is essential for cellular responses to nutrients. These pathways play a critical role in cell growth and metabolism, which are fundamental to the development and function of reproductive cells. These signaling pathways are not directly associated with specific reproductive functions, but their role in key metabolic pathways and energy production may indirectly affect reproductive health and processes in animals (Orozco et al., 2020). Therefore, these findings suggest that these enriched pathways observed in Chuanxiang Black may be associated with better fertility and efficient reproductive processes compared to Landrace pigs.

In addition, miRNAs including those like abu-miR-129, abu-miR-148a-3p, and abu-miR-199-5p, play a significant role in various reproductive processes in pigs. They are involved in processes such as embryo-maternal communication during the peri-implantation period. These small non-coding RNAs help to regulate the expression of genes involved in crucial pathways for successful implantation and early pregnancy stages. The KEGG enrichment analysis of differential miRNAs revealed that Chuanxiang Black pigs showed upregulation of signaling pathways (cAMP signaling pathways, calcium signaling pathways), signaling molecules and interaction (ECM-receptor interaction), endocrine system (aldosterone synthesis and secretion, and GnRH secretion). These pathways indicate the role of specific miRNAs in pigs and provide insights into fertility, spermatogenesis, and overall reproductive health​ (Kaczmarek et al., 2020). They also regulate sperm motility and morphology, which is essential for male fertility. Similar to miRNAs, enrichment of differential circRNAs revealed involvement in signaling pathways (cGMP-PKG signaling, Rap1 signaling, oxytocin signaling, and glucagon signaling), parathyroid hormone synthesis, secretion and action, fatty acid metabolism, focal adhesion, and ECM-receptor interaction. It is well-established that various signaling pathways such as MAPK, AMPK, and TGF-β/Smad signaling play critical roles during spermatogenesis (Lie et al., 2013; Chen and Liu, 2015; Ni et al., 2019). In addition, key signaling pathways identified in the present study have been previously reported to have a crucial role in male spermatogenesis, particularly PI3k/AKT signaling pathway (Khan et al., 2002; Meroni et al., 2002), MAPK signaling pathway (Qi et al., 2014), Rap1 signaling (Aivatiadou et al., 2007) and cAMP/PKA signaling (Santos and Kim, 2010). These signaling pathways dynamically control various steps and processes involved in spermatogenesis and any perturbation in these pathways can result in immature or abnormal spermatozoa subsequently leading to male infertility (Aivatiadou et al., 2007).

Collectively our findings indicate that structural genes regulated by miRNAs and circRNAs as well as differential accumulation of metabolites associated with key pathways including signal transduction (PI3k/AKT signaling, cGMP-PKG signaling, and MAPK signaling), fatty acid metabolism, and signaling molecules control the various physiological networks involved in the reproductive process, spermatogenesis, and sperm quality in pigs.

## 5 Conclusion

Transcriptomic and metabolomic profiling identified differentially expressed genes and several differentially accumulated metabolites between the Chuanxiang Black and Landrace breeds. Our results showed that DEGs and DAMs were involved in reproductive pathways, especially those involved in spermatogenesis. Major pathways identified included signal transduction (PI3K-Akt and MAPK signaling pathways), lipid metabolism, and cytokine-cytokine receptor interactions. These pathways are well known for their critical role in spermatogenesis, sperm maturation, and testicular endocrine function. Our study highlighted the influence of circRNAs and miRNAs in regulation of reproductive processes, underscoring the complexity and intricacy of genetic and metabolic factors governing reproductive efficiency in Chuanxiang Black pigs compared to the Landrace breed. Further studies are needed to provide insights into the regulatory networks and molecular mechanisms underlying the interactions between differentially expressed circRNAs/miRNAs and DEGs that regulate spermatogenesis.

## Data Availability

The datasets presented in this study can be found in online repositories. The names of the repository/repositories and accession number(s) can be found in the article/Supplementary Material.

## Supplementary material

SUPPLEMENTARY FIGURE S1 Density distribution of the expression level of all genes. Distribution of FPKM densities. The x-axis represents the log2 of the FPKM value increased by 1. The y-axis represents the sample density.

SUPPLEMENTARY FIGURE S2 Distribution of differential circRNA in both pig breeds.

SUPPLEMENTARY FIGURE S3 Clustering of samples of both breeds based on FPKM values.
