# Supplementary figures and images for: Combined metabolome and transcriptome analysis provides molecular insights into reproductive process in Chuanxiang Black and Landrace pigs
Source: Front Genet. 2025 Feb 28;16:1501876. doi: 10.3389/fgene.2025.1501876 (PMC11906663; doi:10.3389/fgene.2025.1501876)

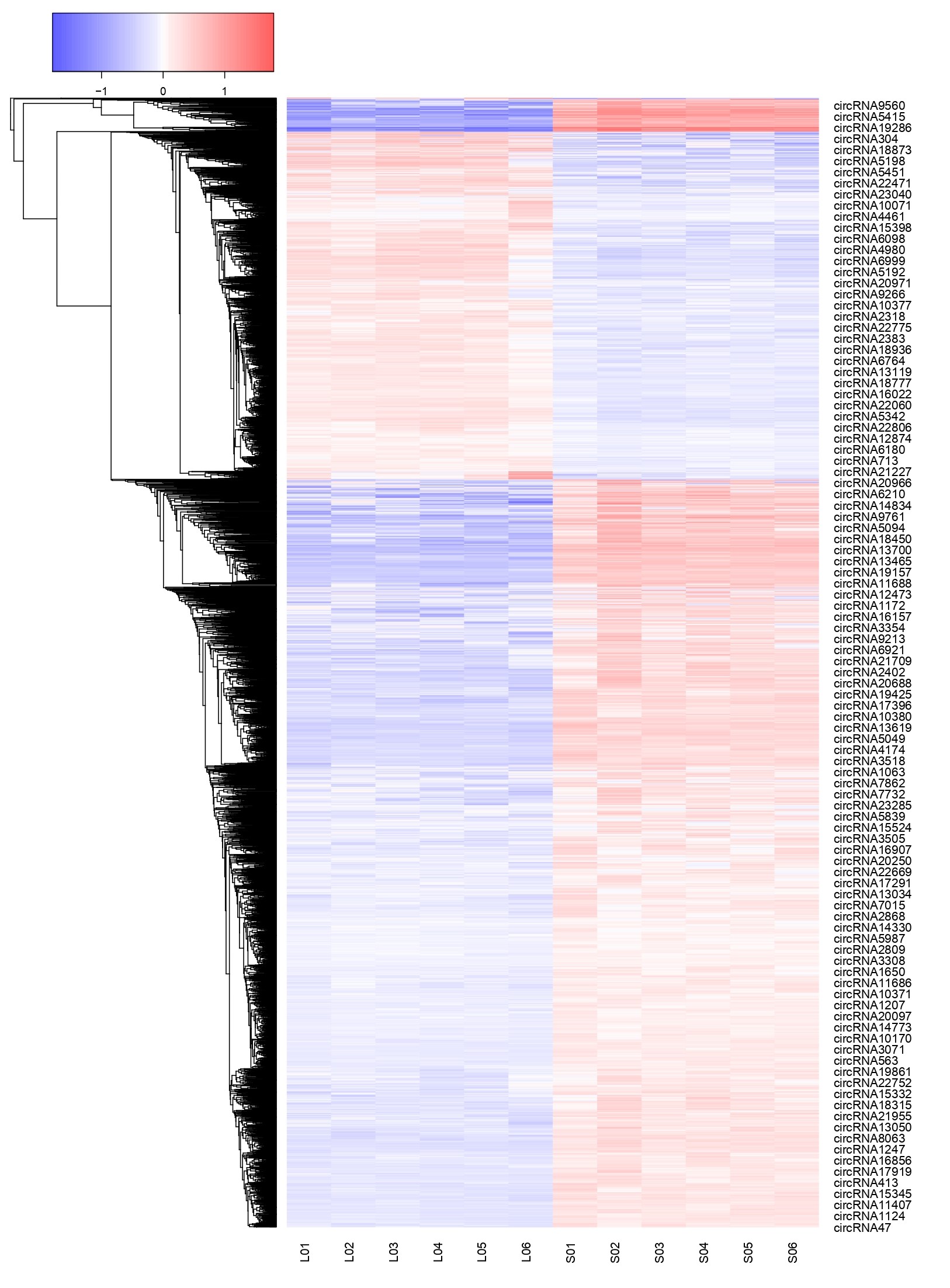

Supplement: Supplementary file 1 [file Image3.jpeg]

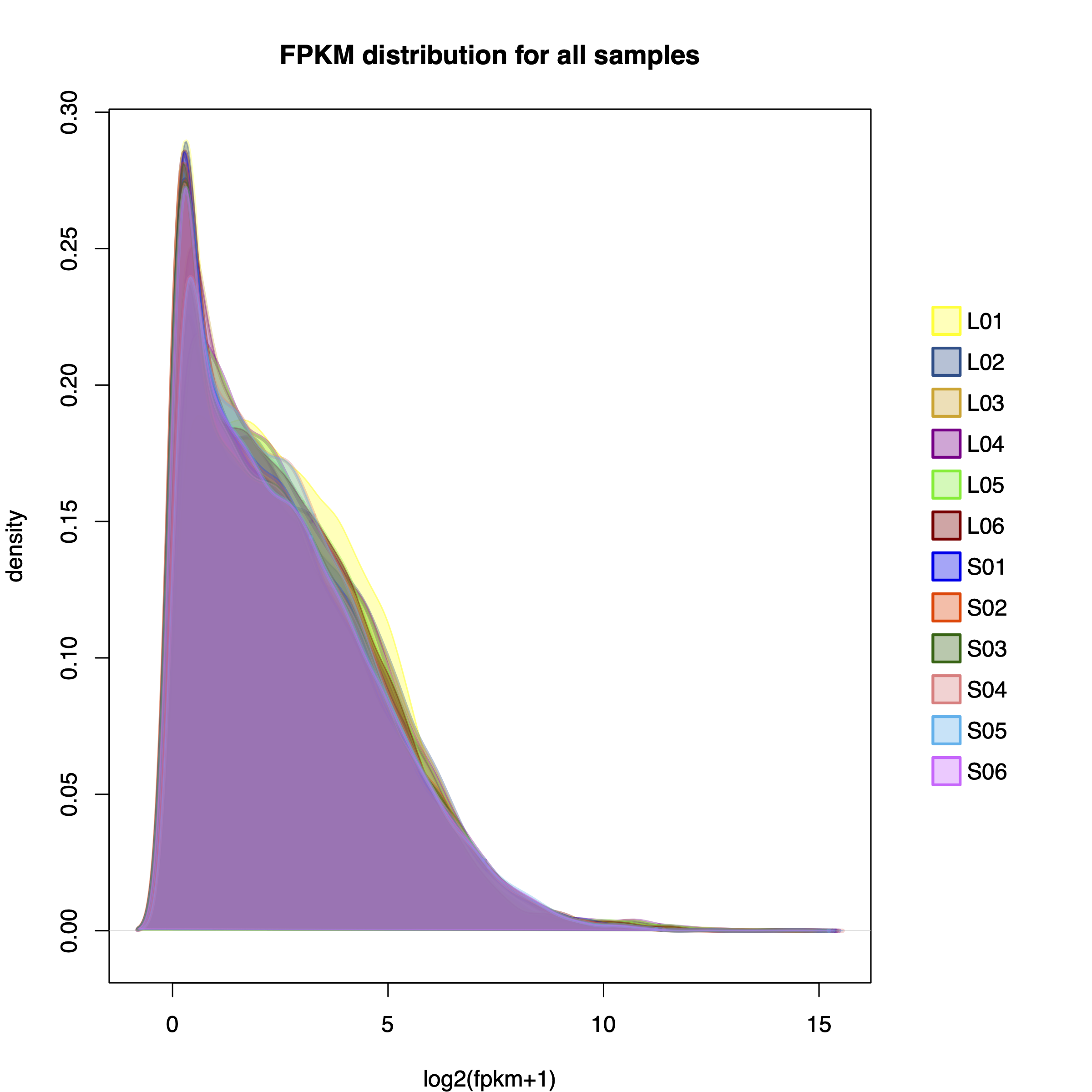

Supplement: Supplementary file 2 [file Image1.tiff]

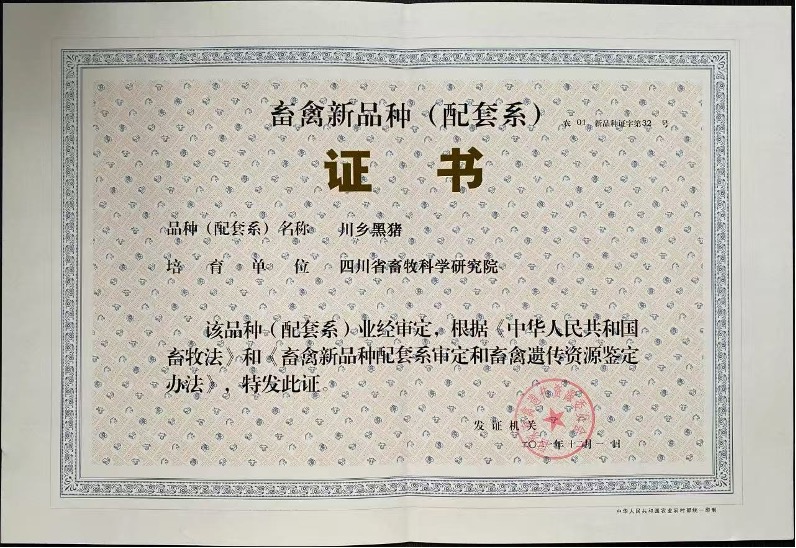

Supplement: Supplementary file 3 [file Image4.jpeg]
